# MRE Encapsulating MRG: Synergistic Improvement in Modulus Tunability and Energy Dissipation

**DOI:** 10.3390/nano15131031

**Published:** 2025-07-03

**Authors:** Mi Zhu, Wang Li, Qi Hou, Yanmei Li

**Affiliations:** 1 College of Artificial Intelligent, Chongqing University of Technology, Chongqing 400050, China; zhumi@cqut.edu.cn; 2 Chongqing Jianshe Industry (Group) Co., Ltd., Chongqing 400054, China; li.wang@cqu.edu.cn; 3 Qinghai Metrological Verification and Testing Institute, Xining 810017, China; qhjlhouqi@163.com

**Keywords:** magnetorheological elastomer (MRE), magnetorheological gel (MRG), modulus tunability, energy dissipation, dynamic response time

## Abstract

Traditional magnetorheological elastomers (MREs) often suffer from limited modulus tunability and insufficient energy dissipation, which restrict their applications. This study prepared a novel composite material by an MR gel (MRG) embedded within the MRE, called the MRE encapsulating MRG, to synergistically enhance these properties. Annular and radial MRE encapsulating MRG configurations were fabricated using 3D-printed molds, and their dynamic mechanical performance was characterized under varying magnetic fields (0–1 T) via a rheometer. The results revealed that the composite materials demonstrated significantly improved magnetic-induced modulus and magnetorheological (MR) effects compared to conventional MREs. Specifically, the annular MRE encapsulating MRG exhibited a 238.47% increase in the MR effect and a 51.35% enhancement in the magnetic-induced modulus compared to traditional MREs. Correspondingly, the radial configuration showed respective improvements of 168.19% and 27.03%. Furthermore, both the annular and radial composites displayed superior energy dissipation capabilities, with loss factors 2.68 and 2.03 times greater than those of pure MREs, respectively. Dynamic response tests indicated that composite materials, particularly the annular MRE encapsulating MRG, achieve faster response times. These advancements highlight the composite’s potential for high-precision damping systems, vibration isolation, and adaptive control applications.

## 1. Introduction

As an important branch of intelligent materials, the magnetorheological elastomer (MRE) possesses excellent mechanical properties and a rapid, reversible, magnetically-enhanced effect [1,2,3], and it has attracted widespread attention from researchers both domestically and internationally. Compared to the traditional magnetorheological fluid, gel, and plastic, the MRE can effectively avoid issues related to the sedimentation and agglomeration of magnetic particles, significantly improving the stability and load-bearing capacity of the materials and their structures [4,5]. This unique advantage allows MREs to exhibit broad application prospects in fields such as intelligent vibration reduction systems [6,7,8,9], flexible sensing/driving [10,11,12,13,14], wearable electronic devices [15,16], and soft robotics [17,18].

The working mechanism of the MRE primarily relies on the dispersion of magnetic particles within the elastic matrix and their interactions under an applied magnetic field, achieving modulus tuning. However, the magnetorheological effect (MR effect) is limited by the particle–matrix interface and its transmission efficiency, often resulting in a limited range of adjustable modulus and insufficient energy dissipation capability (low loss factor), thereby constraining MREs’ application in high-precision damping devices and intelligent sensing, among other areas.

The modulus tunability and energy dissipation performances are crucial properties for the application of MREs, and substantial research has been carried out to enhance them. Some studies have sought to alter the zero-field modulus and magnetoelastic modulus of MREs by changing the polymer matrix, including using silicone rubber [19,20], polydimethylsiloxane (PDMS) elastomers [21,22], polyurethane elastomers [23,24], natural rubber [25,26], ethylene/acrylic elastomers [27,28], waste tire rubber [29], ethylene-propylene-diene monomer (EPDM) rubber [30,31], and others. However, enhancing the MR effect of MREs by modifying the matrix generally comes at the cost of sacrificing the material strength or damping properties. Thus, researchers have proposed various methods, such as particle modification [32,33,34,35], matrix cross-linking [36,37], and doping with synergistic additives [38,39] to gradually enhance the performance of the MRE. To further improve its dynamic properties, some researchers have suggested the fabrication of composite MRE materials. Gong et al. [40] developed composite materials using magnetorheological fluid (MRF) or magnetorheological gel (MRG) combined with the MRE, which increased the materials’ loss factor, and the sealing performance of their composites was poor. Wang et al. [41] prepared a magnetorheological fluid-elastomer (MRF-E) that enhanced the damping characteristics of the application devices, but their solid matrix was a polyurethane elastomer, which does not exhibit magnetorheological effects and cannot further broaden its modulus tunability. Bastola et al. [42,43] utilized 3D-printing technology to develop shape-tunable MREs, effectively improving the materials’ modulus tunability; however, they lost some damping capacity, and the thickness of the produced materials was excessive.

Due to the remarkable field-induced internal reorganization performance of MRG [44], this paper develops a novel composite material of the MRE encapsulating MRG to synergistically improve the modulus tunability and energy dissipation of MREs. First, annularly and radially arranged MRE encapsulating MRGs were prepared based on 3D-printed molds. Next, a rheometer was used to characterize the dynamic mechanical properties of the materials, including storage modulus, loss factor, and dynamic magnetic-induced response time characteristics. Finally, the test results were analyzed, and a comparative analysis of the developed composite materials and the traditional MRE was conducted.

This paper is structured as follows: Section 1 provides an introduction to MREs. Section 2 details the preparation and characterization of the MRE encapsulating MRG samples. Section 3 presents the experimental results and discussion of the MRE encapsulating MRG samples compared to conventional MREs. Finally, Section 4 summarizes the conclusions of the study.

## 2. Materials and Methods

### 2.1. Preparation of MRE Encapsulating MRG Samples

#### 2.1.1. Raw Materials

Two-component (A and B) room temperature vulcanizing silicone rubber (SR, type: E605, Hardness: 5HA), provided by Shenzhen Hongyejie Technology Co., Ltd, Shenzhen, China. Carbonyl iron powders (CIPs, type: CN, particle size: 1~8 μm, purity ≥ 99.5%), provided by BASF Corporation, Ludwigshafen, Germany. Diphenylmethane diisocyanate (MDI: 4, 4 ≈ 50%, 2, 4 ≈ 50%), purchased from Yantai Wanhua Polyurethanes Co., Ltd., Yantai, China. Castor oil (CO), purchased from Sinopharm Chemical Reagent Co., Ltd., Shanghai, China.

#### 2.1.2. Preparation of MRE and MRG Mixtures

MRE mixture: The CIPs were first mixed with component A of SR in a beaker, and they were fully stirred for 20 min at room temperature. Next, component B of SR was added to the mixture, with vigorous stirring at a rotation rate of 500 rpm by using an electric blender (mass ratio of A to B is 1:1), obtaining a homogeneously dispersed MRE mixture (the mass fraction of CIPs was 70%). The detailed preparation process can be found in our previous work [34,45].

MRG mixture: The CO was preheated at 80 °C in a vacuum drying oven for 10 min and then mixed with the CIPs. Next, the MDI was added to the mixture, with vigorous stirring at 500 rpm for 20 min (mole ratio of MDI to CO is 10:1), obtaining a homogeneously dispersed MRG mixture (the mass fraction of CIPs was 50%). The detailed steps can also be found in our previous work [46,47].

#### 2.1.3. Preparation of Composite Samples

**Figure 1:**
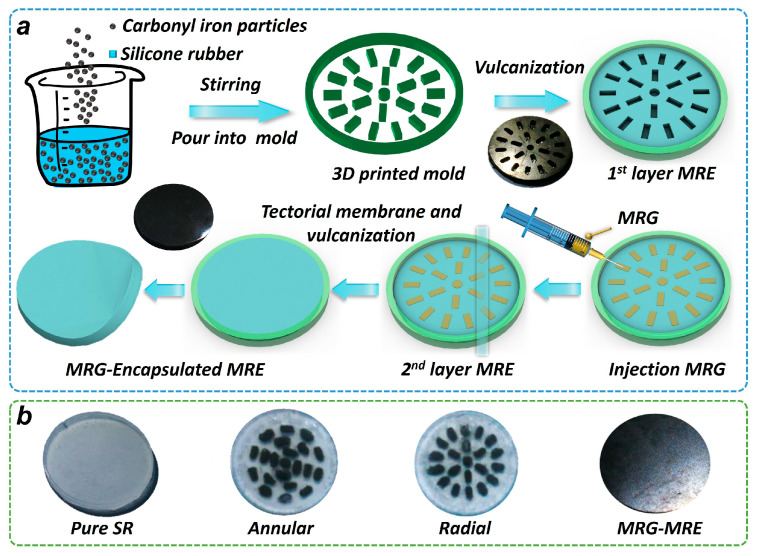
Preparation of MRE encapsulating MRG. (**a**) Preparation process. (**b**) Physical picture of samples.

The fabrication process of the MRE encapsulating MRG samples is shown schematically in Figure 1a. Firstly, a 3D-printed mold was printed via stereolithography (SLA) with a photopolymer resin, forming annular and radial structures to regularize the following MRG distribution. Subsequently, the MRE mixture was injected into a 3D-printed mold and cured at room temperature to form the first layer of the MRE. Next, the rod-shaped and rectangular molds were removed, and the MRG mixture was injected into grooves using a syringe. After the MRG injection, the second MRE layer was added before full vulcanization. Inter-layer covalent bonding was achieved through co-curing at 60 °C, ensuring seamless integration. Finally, an MRE encapsulating MRG with a diameter of 20 mm and a thickness of 2 mm was prepared; the entire process occurs without an additional magnetic field for pre-structuring. Three samples are ultimately prepared: the original MRE, annular MRE encapsulating MRG, and radial MRE encapsulating MRG, which are depicted in the last image in Figure 1b. The internal structure of the composite MRE, pure SR, and annular and radial structure MRG based on the SR is also prepared, as depicted in the first three images of Figure 1b.

It is worth noting that, as Figure 1b shows, there is 1 central cylinder (1.6 mm diameter and 1.1 mm height) and 20 cuboids (2.5 mm length, 1 mm width, and 1.1 mm height) used for embedding the MRG. The diameter of this total composite sample is 20 mm, and its thickness is 2 mm. Therefore, the MRG volume fraction was 8.75% for both the annular and radial configurations, calculated as $V_{MRG}/\left(V_{MRE}+V_{MRG}\right)$, where $V_{MRG}$ and $V_{MRE}$ are the volumes of MRG and MRE in the composite sample, respectively.

### 2.2. Characterization of MRE Encapsulating MRG Samples

**Figure 2:**
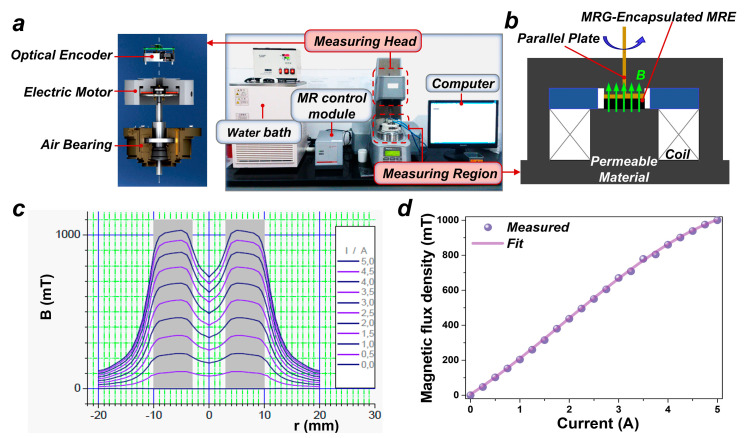
Rheometer test system. (**a**) Main system. (**b**) Measuring region. (**c**) Magnetic field distribution in test area. (**d**) Relationship between magnetic flux density and control current.

The dynamic mechanical properties of the samples are measured by an MCR301 rotary rheometer (Anton Paar Germany GmbH, Ostfildern, Germany), as shown in Figure 2. The test system comprises a water bath, computer, measuring head, measuring region, and an MR control module, as illustrated in Figure 2a. The water bath functions in conjunction with the program to achieve liquid circulation temperature control, providing a stable and controllable temperature environment for the testing of the MRE samples. The computer is mainly utilized for program control during the sample testing process. The measuring head is one of the key parts of the rheometer, comprising an electric motor, optical encoder, and air bearing. Thereinto, the electric motor is primarily used to apply or measure the shear stress on the MRE samples, while a linear optical encoder accurately computes the shear strain during the test. The air bearings ensure that the samples experience no mechanical friction throughout the testing process, thereby guaranteeing an extremely low error. During the testing, the MRE sample is positioned in the measuring region (a diameter of 20 mm and a thickness of 2 mm), which is located between the parallel plate rotor and the substrate, with the upper part of the parallel plate rotor connected to the measuring head of the rheometer; a schematic of the MRE testing region is shown in Figure 2b. The MR control module, the most crucial part of the rheometer, is specifically employed to regulate the magnetic field within the measuring region. When the control current is applied to the system coils via the MR module, a uniform magnetic field is produced in the MRE testing area. Currents ranging from 0 to 5A are applied; the distribution of the magnetic flux intensity in the measuring region is depicted in Figure 2c [48]. The relationship between the average magnetic flux intensity and the current is fitted, as shown in Figure 2d, with the fitted equation as follows:

(1) $$B=0.45142+184.48106\times I+25.48947\times I^{2}-4.47582\times I^{3}$$

where B is the magnetic flux density and its unit is mT, and I is the excitation current and its unit is A. The fitted results will be utilized to convert between the current and magnetic field values in subsequent test results.

This study primarily encompasses magnetic field scanning tests and dynamic magneto-responsive time tests. The magnetic field scanning tests aim to obtain the storage modulus, loss modulus, and loss factor characteristics of the MRE samples under varying magnetic fields. In the testing process, the current is varied from 0A to5A, which corresponds to a magnetic field variation from 0 mT to 1000 mT. The testing temperature is maintained at 25 °C, with a frequency of 10 Hz and the strain fixed at 0.1%. The dynamic magneto-induced response time tests are designed to assess the temporal changes in the dynamic response of the MRE samples under transient magnetic field conditions. During this phase, the temperature remains set at 25 °C, the frequency at 10 Hz, and the strain at 0.1%. The transient excitation current during testing is configured as 0A to 5A to 0A, with the total testing duration established at 250 s. Each test was conducted three times, with the average of the three trials recorded as the final result.

## 3. Results and Discussion

### 3.1. Storage Modulus Results and Analysis

**Figure 3:**
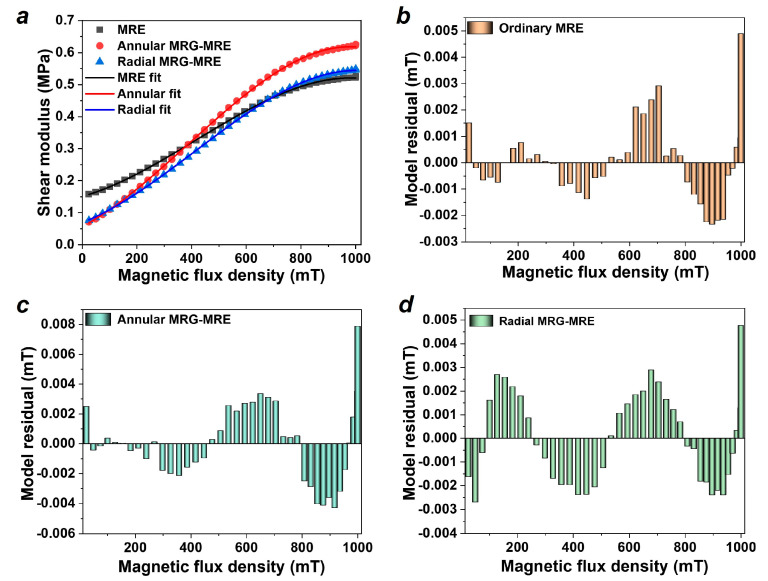
Shear modulus results (test frequency 10 Hz, strain 0.1%, temperature 25 °C, current 0–5 A). (**a**) Test and model fitting results. (**b**–**d**) Residual histogram of model.

The results of the shear storage modulus of the MRE samples are shown in Figure 3a. To describe the relationship between the modulus and the magnetic field, a macroscopic phenomenological constitutive model is established as

(2) $$G\left(B\right)=G_{0}+\alpha B+\beta B^{2}+\gamma B^{3}$$

where $G_{0}$ is the initial shear modulus of MRE, B is the magnetic flux intensity, and α, β, and γ are the magnetic field sensitivity coefficients.

The fitting results are also depicted in Figure 3a, and the residual histogram of the model on the MRE, annular, and radial MRE encapsulating MRGs is shown in Figure 3b–d. It can be seen that the model fits the test results well, and their residuals are all relatively small (no more than 0.008). The parameters fitted from the model are shown in Table 1. It can be observed that the initial shear modulus $G_{0}$ of the MRE encapsulating MRG is lower than that of the ordinary MRE. This is because the MRE encapsulating MRG is composed of two materials; its shear modulus is listed in Formula (3). The modulus of the gel-like MRG is less than that of the solid MRE, resulting in a lower initial modulus $G_{0}$ for the composite material compared to that of the MRE.

(3) $$G=G_{MRE}V_{MRE}+G_{MRG}V_{MRG}$$

where $G_{MRE}$ and $G_{MRG}$ are the shear storage modulus of MRE and MRG, respectively. $V_{MRE}$ and $V_{MRG}$ are the volume fractions of MRE and MRG, respectively.

**Figure 4:**
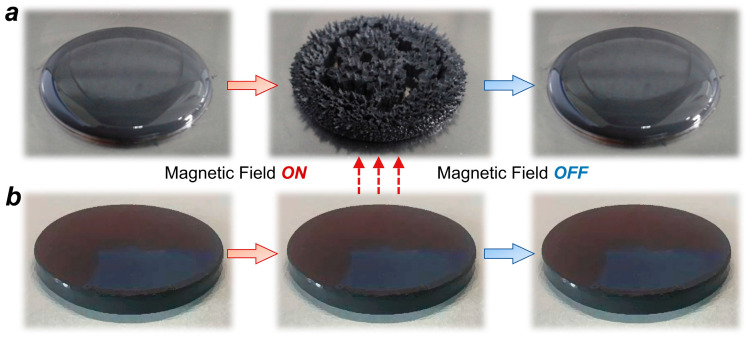
Macroscopic representation after applying/removing magnetic field. (**a**) Phase transition characteristic of MRG. (**b**) MRE without visible phase transition.

After applying a magnetic field, the CIPs move freely inside the MRG’s gel matrix and form chain or columnar structures. Once the magnetic field is removed, this structure returns to the initial uniformly dispersed state. Macroscopically, the MRG shows significant phase transition characteristics under the influence of a magnetic field, as shown in Figure 4a. In contrast, the solid matrix of the MRE exhibits strong binding capabilities under a magnetic field; the CIPs cannot move freely within the matrix. Instead, the interaction forces between the particles or between the particles and the matrix are enhanced. Upon the removal of the magnetic field, these interaction forces rapidly diminish until they disappear. Macroscopically, the MRE does not exhibit a visible phase transition under the influence of the magnetic field, as shown in Figure 4b.

**Figure 5:**
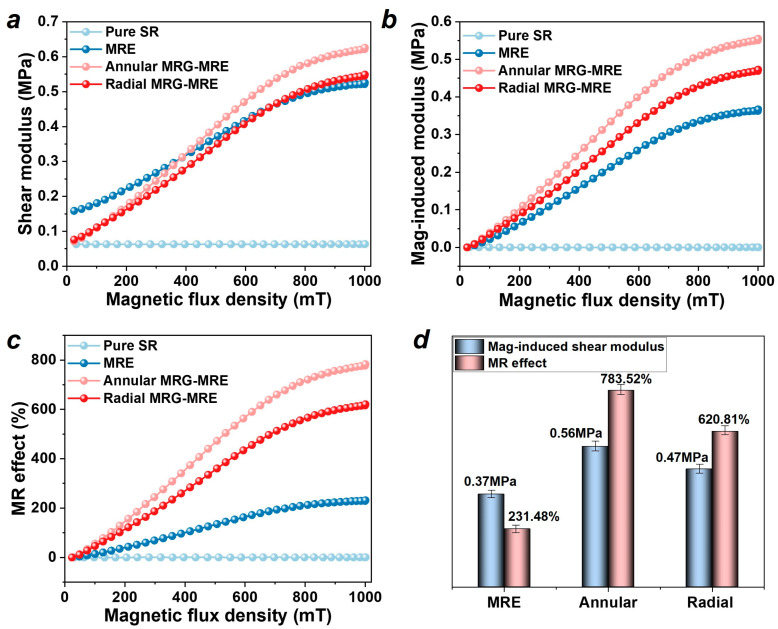
Magnetic-induced modulus and MR effect results (test frequency 10 Hz, strain 0.1%, temperature 25 °C, current 0–5 A). (**a**) Shear modulus. (**b**) Magnetic-induced modulus. (**c**) MR effect. (**d**) Comparison of maximum magnetic-induced modulus and MR effect.

Therefore, after applying the magnetic field, the CIPs inside the annular and radial MRG rapidly form chains, generating external forces that promote a rapid increase in the shear modulus. The magnetic-induced modulus of samples is defined as the magnetic-induced change of shear storage modulus $\Delta G=G-G_{0}$, and the MR effect is defined as the relative change of shear storage modulus $MR_{effect}=\Delta G/G_{0}\times 100\%$. Compared to the ordinary MRE, the MRE encapsulating MRG exhibits a higher magnetic-induced modulus and MR effect (as shown in Figure 5a–d). The results of the maximum magnetic-induced modulus and MR effect of all samples are listed in Table 2 (containing standard deviations (*n* = 3), the relative standard deviation < 5% for all reported values). Among them, the maximum magnetic-induced moduli of annular and radial MRE encapsulating MRGs are 0.56 MPa and 0.47 MPa, respectively, which represent increases of 51.35% and 27.03% compared to the 0.37 MPa of the ordinary MRE. Furthermore, compared to the maximum MR effect of the ordinary MRE at 231.48%, the annular and radial MRE encapsulating MRGs achieved effects of 783.52% and 620.81%, respectively, representing increases of 238.47% and 168.19%. The annular MRE encapsulating MRG achieved the highest magnetic-induced modulus and MR effect, mainly due to the more concentrated annular MRG inside the MRE, which can form a denser chain of particles under the influence of the magnetic field, thus generating a greater force and MR effect. The modulus tunability of the composite MRG-MRE material proposed in this paper has been effectively enhanced, and it can be applied to broadband vibration isolation devices and control systems in the future.

### 3.2. Loss Modulus Results and Analysis

**Figure 6:**
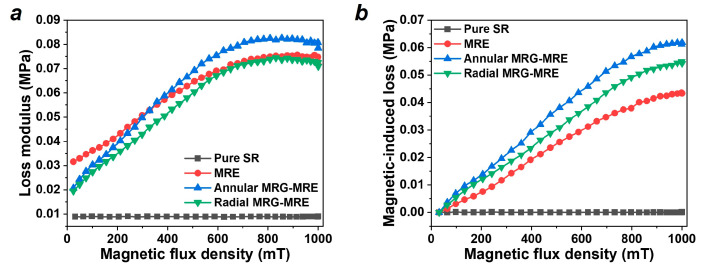
Loss modulus results (test frequency 10 Hz, strain 0.1%, temperature 25 °C, current 0–5 A). (**a**) Loss modulus. (**b**) Magnetic-induced loss modulus.

The results of the loss modulus and magnetic-induced loss modulus of the MRE samples are shown in Figure 6, which illustrates the energy dissipated in the form of thermal energy during the dynamic loading process, indicating the damping capacity of the material. It can be seen that the annular MRE encapsulating MRG achieved the highest magnetic-induced loss modulus.

**Figure 7:**
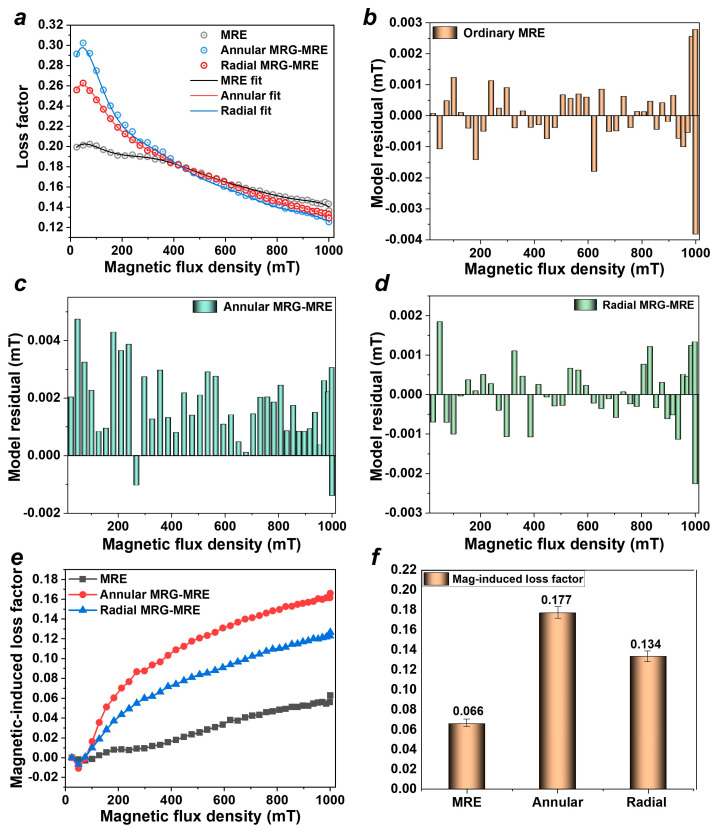
Loss factor results (test frequency 10 Hz, strain 0.1%, temperature 25 °C, current 0–5 A). (**a**) Test and model fitting results of loss factor. (**b–d**) Residual histogram of model. (**e**) Magnetic-induced loss factor. (**f**) Magnetic-induced loss modulus and loss factor.

Similarly, the loss factor of the MRE is the ratio of the loss modulus to the storage modulus, characterizing the energy dissipation efficiency (damping efficiency) of the material. This factor directly affects the magnitude of the resonance peak in MRE vibration isolation systems, making it a very important assessment parameter in damping applications. Figure 7a depicts the loss factor of each sample, showing a general decreasing trend as the magnetic field increases. To describe this trend, a polynomial model is established as follows:

(4) $$D\left(B\right)=D_{0}+a_{1}B+a_{2}B^{2}+a_{3}B^{3}+a_{4}B^{4}+a_{5}B^{5}+a_{6}B^{6}$$

where $D_{0}$ is the initial loss factor of MRE, B is the magnetic flux intensity, and $a_{1}$~$a_{6}$ are the magnetic field sensitivity coefficients.

The fitting results are shown by the solid lines in Figure 7a, and the residual histogram of the model fitting is displayed in Figure 7b–d. It can be observed that this model effectively describes the trend of the MRE’s loss factor with changes in the magnetic field, and the fitting parameters are listed in Table 3. Figure 7e presents the magnetic-induced loss factor of the materials, indicating that the magnetic-induced loss factor of the composite materials is higher than that of the ordinary MRE. Among them, the annular MRE encapsulating MRG has the highest magnetic-induced loss factor at 0.177, followed by the radial configuration at 0.134, as shown in Figure 7f. Compared to the ordinary MRE’s 0.066, the annular and radial MRE encapsulating MRGs have increased 2.68 and 2.03 times, respectively.

The overall damping property of the MRE encapsulating MRG can be simplified to the following:

(5) $$D=D_{CMRE}+D_{IMRE}+D_{CMRG}+D_{IMRG}$$

where $D_{CMRE}$ and $D_{CMRG}$ are the intrinsic damping capacity of MRE and MRG, respectively. $D_{IMRE}$ and $D_{IMRG}$ are the interface damping of MRE and MRG, respectively, which is mainly caused by the internal friction between the particles and polymer material where the relative motion takes place.

In the composite material, the MRG, as a gelling material, has an intrinsic damping characteristic $D_{CMRG}$ greater than that of MRE $D_{CMRE}$. Furthermore, after applying a magnetic field, the magnetic particles in the MRG will align in a chain-like arrangement, increasing the mutual friction between the particles and the matrix, thus making $D_{IMRG}$ greater than $D_{IMRE}$. In summary, the damping characteristics of the MRE encapsulating MRG are superior to those of the ordinary MRE. Additionally, the annular MRE encapsulating MRG achieved the highest magnetic-induced loss factor, largely due to the more concentrated annular MRG within the MRE, which can increase the concentrated relative motion and friction between the particles and the matrix, thereby enhancing its damping dissipation capacity.

### 3.3. Dynamic Magnetic-Induced Response Time Results and Analysis

**Figure 8:**
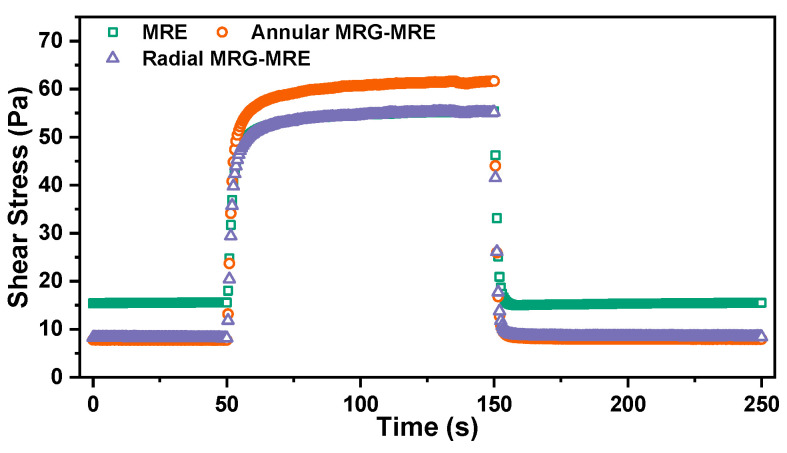
Dynamic magnetic-induced response under step current of 5A (test frequency 10 Hz, strain 0.1%, temperature 25 °C, transient excitation current 0 A-5 A-0 A).

The study of the dynamic response time characteristics of the MRE mainly focuses on the material’s dynamic mechanical performance under dynamic conditions (oscillating sinusoidal strain). By applying and removing a step current/magnetic field, the response over a long time scale is evaluated. Under the excitation of a 5 A step current, the response time curve of the sample’s shear stress characteristics is shown in Figure 8. After the current is applied (t = 50 s), the stress gradually stabilizes over time; after the current is removed (t = 150 s), it rapidly approaches a stable state.

A double exponential decay function is used to describe the rising and falling response time processes of the MRE.

(6) $$\sigma \left(t\right)=A_{0}+A_{1}e^{-t/\tau_{1}}+A_{2}e^{-t/\tau_{2}}$$

where the constants $A_{0}$, $A_{1}$, and $A_{2}$ are parameters in the model. $\tau_{1}$ and $\tau_{2}$ represent the fast and slow response time constants of the MRE under the influence of the step current, corresponding to the early rapid change phase and the late steady-state phase of the dynamic response process, respectively.

**Figure 9:**
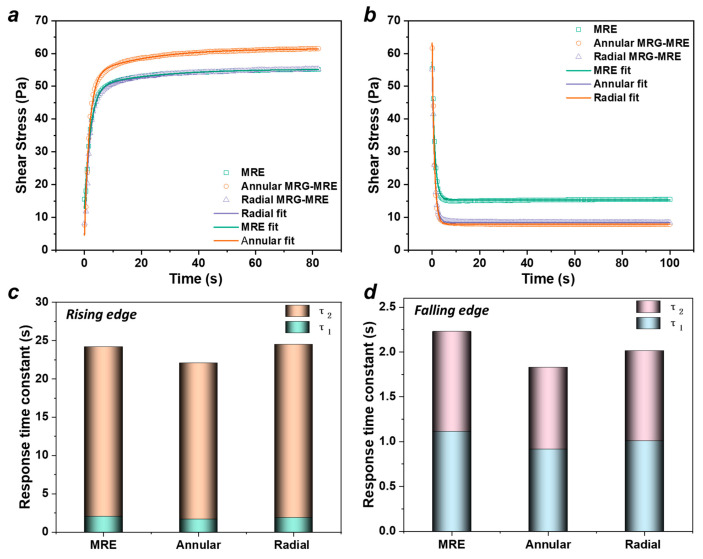
(**a**,**b**) Response fitting results on rising and falling edges under step current of 5A. (**c**,**d**) Characteristic response time constants of three materials.

Using Equation (6) to fit the magnetic-rising and falling process shown in Figure 8, the fitting results are illustrated in Figure 9a,b, with the fitting parameter results presented in Table 4 and Table 5. The characteristic response time constants of the three materials are shown in Figure 9c,d. It can be observed that the magnetic-falling process is faster than the rising process, which is mainly due to the rapid disappearance of the interparticle interaction forces within the MRE once the current is removed, allowing the particle chain structure of the MRG to quickly return to a dispersed state. Additionally, regardless of whether it is the magnetic-rising or falling process, the composite MRE encapsulating MRG material demonstrates a faster response compared to the conventional MRE material. This is primarily because, under the influence of the magnetic field, the MRG in the composite material more readily forms “magnetic pathways,” thus accelerating the dynamic characteristic response. Notably, the characteristic response time constants of the annular composite material are the smallest, with rise times of 1.7036 s and 20.3842 s and fall times of 0.9158 s and 0.9126 s, respectively. This is attributed to the denser distribution of the MRG within the annular structure, which allows for greater magnetic interaction forces among the internal particles under the magnetic field, thereby more easily overcoming the internal magnetic resistance of the MRE to achieve dynamic equilibrium.

## 4. Conclusions

This study presents a novel strategy for enhancing the modulus tunability and energy dissipation of MREs through the encapsulation of the MRG. By fabricating annular and radial configurations of MRE encapsulating MRGs using 3D-printed molds, the research demonstrates significant synergistic improvements in the dynamic mechanical properties compared to conventional MREs. The experimental results revealed that the annular MRE encapsulating MRG exhibited a 51.35% increase in the magnetic-induced modulus and a 238.47% improvement in the MR effect compared to the traditional MRE, while the radial configuration showed 27.03% and 168.19% enhancements, respectively. Furthermore, the composite materials demonstrated exceptional energy dissipation, with loss factors 2.68 and 2.03 times higher than conventional MREs. The dynamic magnetic-response tests highlighted a faster dynamic response time in the composite MREs, particularly in the annular MRE encapsulating MRG, due to the efficient "magnetic pathway" formation. These advancements position MRE encapsulating MRGs as promising candidates for high-precision damping systems, adaptive vibration isolation, and intelligent control applications. Future work will explore the long-term stability and integration into vibration isolation devices to fully leverage the material’s tunable and dissipative capabilities.

## Figures and Tables

**Table 1:** The fitting parameters of the shear modulus model on all samples.

Parameters	$G_{0}$ (MPa)	α (MPa/mT)	β (MPa/mT^2^)	γ (MPa/mT^3^)
Ordinary MRE	0.1509	2.3936 × 10^−4^	6.6424 × 10^−7^	−5.3392 × 10^−10^
Annular MRG-MRE	0.0571	4.4592 × 10^−4^	8.4374 × 10^−7^	−7.2881 × 10^−10^
Radial MRG-MRE	0.0694	3.3099 × 10^−4^	7.6016 × 10^−7^	−6.1639 × 10^−10^

**Table 2:** The maximum magnetic-induced modulus and the MR effect of all samples.

Sample	Magnetic-Induced Modulus	MR Effect
Pure SR	/	/
Ordinary MRE	0.3669 ± 0.012 MPa	231.48 ± 8.5%
Annular MRG-MRE	0.5550 ± 0.019 MPa	783.52 ± 26.3%
Radial MRG-MRE	0.4728 ± 0.016 MPa	620.81 ± 21.1%

**Table 3:** The fitting parameters of the loss factor model on all samples.

Parameters	$D_{0}$	$a_{1}$ (mT^−1^)	$a_{2}$ (mT^−2^)	$a_{3}$ (mT^−3^)	$a_{4}$ (mT^−4^)	$a_{5}$ (mT^−5^)	$a_{6}$ (mT^−6^)
Ordinary MRE	0.19	6.27 × 10^−4^	−9.70 × 10^−6^	6.39 × 10^−8^	−2.28 × 10^−10^	4.77 × 10^−13^	−6.02 × 10^−16^
Annular MRG-MRE	0.29	−0.01	2.06 × 10^−4^	−1.51 × 10^−6^	6.20 × 10^−9^	−1.52 × 10^−11^	2.27 × 10^−14^
Radial MRG-MRE	0.25	−0.01	1.77 × 10^−4^	−1.31 × 10^−6^	5.35 × 10^−9^	−1.31 × 10^−11^	1.96 × 10^−14^

**Table 4:** The fitting parameters of the rise edge on all samples.

Parameters	$A_{0}$	$A_{1}$	$A_{2}$	$\tau_{1}$	$\tau_{2}$
Ordinary MRE	55.2517	−36.7373	−5.7108	2.0620	22.1336
Annular MRG-MRE	61.5137	−48.6466	−8.2622	1.7036	20.3842
Radial MRG-MRE	55.3081	−44.0505	−6.2676	1.8838	22.6456

**Table 5:** The fitting parameters of the fall edge on all samples.

Parameters	$A_{0}$	$A_{1}$	$A_{2}$	$\tau_{1}$	$\tau_{2}$
Ordinary MRE	15.2875	32.4476	9.7183	1.1140	1.1141
Annular MRG-MRE	7.9952	44.5825	10.6378	0.9158	0.9126
Radial MRG-MRE	8.4923	44.4016	3.8252	1.0066	1.0065

## Data Availability

The data that support the findings of this study are available on request from the corresponding author.
